# Identifying priority core habitats and corridors for effective conservation of brown bears in Iran

**DOI:** 10.1038/s41598-020-79970-z

**Published:** 2021-01-13

**Authors:** A. Mohammadi, K. Almasieh, D. Nayeri, F. Ataei, A. Khani, J. V. López-Bao, V. Penteriani, S. A. Cushman

**Affiliations:** 1 Department of Environmental Science and Engineering, Faculty of Natural Resources, University of Jiroft, Jiroft, Iran; 2 Department of Nature Engineering, Agricultural Sciences, Natural Resources University of Khuzestan, Mollasani, Iran; 3 Department of Environmental Sciences, Faculty of Natural Resources, University of Tehran, Karaj, Iran; 4 College of Environment, Karaj, Iran; 5 Khorasan-E Razavi Provincial Office of the Department of the Environment, Mashhad, Iran; 6 Research Unit of Biodiversity (UMIB, CSIC/UO/PA), 33600 Mieres, Spain; 7 USDA Forest Service Rocky Mountain Research Station, 2500 S. Pine Knoll, Flagstaff, AZ USA

**Keywords:** Conservation biology, Ecological modelling

## Abstract

Iran lies at the southernmost range limit of brown bears globally. Therefore, understanding the habitat associations and patterns of population connectivity for brown bears in Iran is relevant for the species’ conservation. We applied species distribution modeling to predict habitat suitability and connectivity modeling to identify population core areas and corridors. Our results showed that forest density, topographical roughness, NDVI and human footprint were the most influential variables in predicting brown bear distribution. The most crucial core areas and corridor networks for brown bear are concentrated in the Alborz and Zagros Mountains. These two core areas were predicted to be fragmented into a total of fifteen isolated patches if dispersal of brown bear across the landscape is limited to 50,000 cost units, and aggregates into two isolated habitat patches if the species is capable of dispersing 400,000 cost units. We found low overlap between corridors, and core habitats with protected areas, suggesting that the existing protected area network may not be adequate for the conservation of brown bear in Iran. Our results suggest that effective conservation of brown bears in Iran requires protection of both core habitats and the corridors between them, especially outside Iran’s network of protected areas.

## Introduction

Large carnivore populations increasingly face threats from habitat loss, fragmentation and isolation worldwide^1–5^. Additionally, large carnivores often reside in and disperse across landscapes characterized by heterogeneous patterns of habitat suitability and mortality risk^6–10^. Existing protected areas often fail to support viable populations of large carnivores in many parts of the world as a result of their large area requirements, low densities and high dispersal abilities^6–11^. Thus, conservation planning for large carnivores requires assessing the efficacy of existing protected areas, prioritizing establishment of new protected areas in strategic locations, and protection of dispersing individuals as they move between these networks of protected areas^12–16^.

One of the approaches to ensure regional large carnivore long-term viability is based on establishing and protecting large core habitat patches, and a network of connectivity linkages, and low-risk areas among them^15,17,18^. Thus, spatially connected networks of core habitat patches often emerges as a priority to reach landscapes of coexistence between humans and large carnivores^8,10,19–22^ on national and even international population level approach^23,24^.

Conservation prioritization depends on accurate assessment of the importance of areas as habitat core areas supporting local populations and also prediction of patterns of connectivity among these core population areas. Habitat suitability modeling and connectivity analyses are foundational to these two critical objectives. In recent years several studies have used multi-scale habitat modeling as the foundation for conservation prioritization^11,22,25–29^, concluding that multi-scale optimization greatly improves prediction of habitat quality^30^.

Connectivity analyses are often based on resistance surfaces extracted from habitat models^25–27^. While recent studies have shown that habitat selection is often a poor proxy for resistance to movement and dispersal^31–33^, and that genetic and movement data should be used when they are available^34^. However, when such data are not extant, as in the case for brown bear in Iran, habitat quality can be used as the basis of predicting landscape resistance to movement^32,33^.

A variety of methods have been used to assess landscape connectivity, including least-cost path modeling^35^, current flow^36^, factorial least-cost path density^37^, resistant kernels^38^ and randomized shortest path algorithms^39^. The factorial least-cost path and cumulative resistant kernel approaches have been used in combination and the complementary information they provide have produced useful conservation tools^9,15,16,18,34,40–43^. In particular factorial least cost path and resistant kernel analysis incorporates scale dependence of dispersal ability, which is critical to produce accurate estimates of synoptic connectivity^40,44^. This combined connectivity modeling approach, coupled with landscape pattern analysis^45^, may provide a useful framework to predict the location of core areas, fracture zones (i.e., where connectivity is attenuated by barriers or cumulative dispersal cost), and movement corridors across a range of dispersal abilities^9,16,22,40^.

The drivers of habitat suitability and connectivity of brown bear (*Ursus arctos* Linnaeus, 1758) populations in Asia are still poorly understood^46–48^. It is known, however, that in western Asia the range of the brown bear has dramatically decreased in recent decades^49^. Small numbers of individuals within isolated sub-populations are currently distributed across Iran, Iraq, Turkey, Georgia, Armenia and Azerbaijan, and have been recently rediscovered in Syria^47,50,51^.

Iran represents the southernmost limit of brown bear global distribution^52,53^ and bears here belong to a unique clade with limited connectivity with other populations^54^. Brown bears in Iran are scattered throughout the mountainous areas of the country^55,56^, and the species is listed as nationally endangered (EN) under criteria C1^57^. The species is categorized as a protected species by the Iranian Department of the Environment and, therefore, bear hunting is illegal in Iran^58,59^. Considering the conservation status of bears in the Middle East^47^, shedding light into the habitat quality and connectivity of brown bear populations in Iran is important to large-scale conservation planning for the species.

In this study, we applied a combination of multi-scale habitat modeling and landscape connectivity analyses to: (1) assess the influence of the spatial scale of environmental variables on habitat selection and identify which variables have the most contribution; (2) identify core habitat patches and corridor paths; (3) assess the representation of the species range in predicted core habitats; and (4) prioritize the predicted core habitats based on probability of connectivity. Such information is crucial to help conservation managers in prioritizing key habitats for brown bears and, more generally, large carnivores, by considering their spatial position and evaluating their role in improving dispersal movement across the landscape.

## Materials and methods

**Figure 1:**
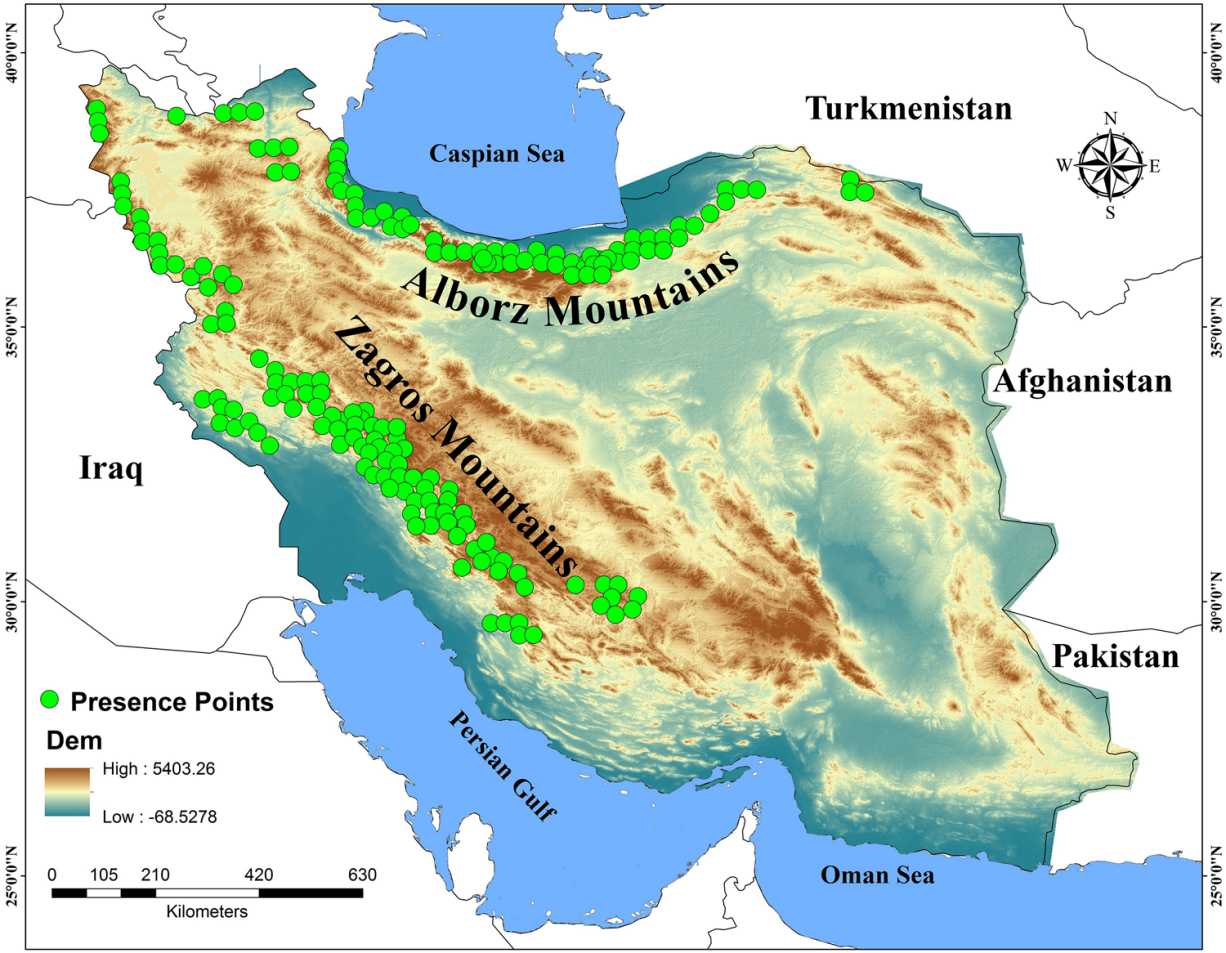
Current distribution with presence points (n = 208) of brown bear in Iran presented on an elevation map.

Iran has two distinct topographic contexts: mountain ranges, which consist principally of the Alborz and Zagros Mountains, which form an arc across northern, western and southwestern Iran, and vast arid plains, mainly in the central and southern parts of Iran (Fig. 1). The brown bear distribution is concentrated in (1) the Zagros Mountain, which ranges from Kurdistan in the west to Fars in the south of Iran, (2) the Alborz mountains from east of the Kopet-Dag Mountain (northeastern Iran), and from southeast to southwest of the Caspian Sea, (3) in the Qara-Daq mountain range, and (4) the Sabalan and Sahand volcanic mountains in the northwestern parts of Iran^59,60^. This study covers the entire range of the brown bear in Iran, covering approximately 1,648,195 km^2^^60^.

### Data collection and spatial filtering

Brown bear occurrence points (n = 208) were obtained from a variety of sources including opportunistic direct observations, signs, mortality records and camera trap photos which were gathered by game rangers of Iranian Department of Environment (DoE) and researches during 2005–2020. Prior to analysis the quality, reliability and precision of all data were evaluated, and only locations with high certainty of identification and location were included. Given the challenges of acquiring data in the Iranian context, we were not able to collect more presence points of brown bears in Iran as this species has not been surveyed systematically in the country.

To reduce spatial autocorrelation in the occurrence points, which can bias model predictions, we spatially filtered points prior to analysis^26,61^. Based on the mean size of male brown bear home ranges in Turkey (83 km^2^)^62^, circles of 5 km–radius were centered on each presence point to exclude proximal points using the Spatially Rarify Occurrence Data tool in the SDMtoolbox^63^. After this spatial filtering we retained a total of 184 presence points in the final dataset used for habitat modeling.

### Environmental variables for habitat modeling

We selected topographic, climatic, land cover and human footprint variables for habitat modeling of the brown bear in Iran based on previously published habitat relationships models for the species^25^. A digital elevation model (DEM) from the 30 m Shuttle Radar Topography Mission (SRTM, downloaded from http://earthexplorer.usgs.gov), was used to calculate slope (using Surface Tool in Spatial Analyst Tools) and surface roughness variables (Geomorphometry and Gradient Metrics toolkit)^64^ in ArcGIS 10.3. The national land-cover map of Iran^65^ was used to create three land use variables: orchard, grassland and forest. A circular focal mean moving window with 2.5 km radius was used to create density maps for each land cover class (orchard, grassland and forest). The 16-day composite MODIS data (MOD13A1 V6 map at 500-m cell size; http://earthexplorer.usgs.gov) was used to calculate the mean normalized difference vegetation index (NDVI) values of the year 2010. Due to the importance of water resources for wildlife^53^, distance from rivers was calculated. Human footprint was represented by several variables, given that distinguishing the effects of different kinds of human perturbation is important (i.e. human population density, human infrastructure and road network)^66^. Out of 19 bioclimatic variables (http://worldclim.org)^67^, we selected six variables that we believed to be most relevant to predicting the distributions of the species: annual mean temperature (BIO1), max temperature of the warmest month (BIO5), min temperature of the coldest month (BIO6), annual precipitation (BIO12), precipitation of warmest month (BIO13) and precipitation of driest month (BIO14).

### Multi-scale species distribution modeling

In addition to the 184 occurrence records, we created 1000 pseudo-absence points randomly placed across the study area outside of the 5 km–radius circles^25^. We used two methods to reduce multicollinearity among variables: (1) the MaxentVariableSelection package^68^ in R^69^ was used to exclude variables by setting a contribution threshold of 1%, regularization multiplier of 1–5 with increments of 0.5 and inter-correlation of 0.7. Variables with the highest area under the curve (AUC) of receiver operating characteristic (ROC) and the lowest Akaike Information Criterion (AIC) were chosen; and (2) the Variance Inflation Factor (VIF) of the dataset was checked using r-package usdm^70^ to exclude variables with VIF > 3^71^.

We used random forest (RF)^72^ to predict habitat suitability for the brown bear, using with a multi-scale approach^73,74^. Multiple-scale modeling with random forest has been shown to outperform other approaches for ecological prediction^17,75^. Six scales (1, 2, 4, 8, 16 and 32 km)^25^ were calculated for each variable using variable radius focal mean analysis. These selected scales span the range of brown bear habitat requirements, from resources within core habitats to the extent of reported home ranges of the species^48^, and correspond to the scale range previously shown to be relevant to large carnivore’s^11,75^ and for brown bears in particular^25^.

For each variable at each scale, we ran univariate RF and the performance of each scale of habitat suitability model was evaluated using AUC and True Statistic Skill (TSS). The scale with the highest performance was chosen for including in a final multivariate optimized model^75,76^. Variable contributions for each model were calculated, and we produced response curves of presence points to the retained predictor variables. All of these analyses were carried out using the Random forest r-package^73^.

### Transforming habitat suitability to landscape resistance

To estimate landscape resistance^77^, we converted the habitat suitability maps to resistance maps using the Eq. 1^78^:

1 $${\text{R}} = 1000^{{( - 1 \times {\text{HS}})}}$$

where R represents the cost resistance value assigned to each pixel and HS represents the predicted habitat suitability derived from the suitability models described above^32,78^. We rescaled the resistance values to a range between 1 and 10 by linear interpolation, such that minimum resistance (Rmin) was 1 when HS was 1, and maximum resistance (Rmax) was 10 when HS was 0^78^.

### Connectivity modeling

We used the universal corridor network simulator (UNICOR)^79^ to create two sets of connectivity predictions including (1) resistant kernels^38^ and (2) factorial least-cost paths^37^. The factorial least-cost path analysis implemented in the UNICOR simulator applies Dijkstra’s algorithm to resolve the single-source shortest path issue from every mapped species occurrence location on a landscape to every other occurrence location^79^. The analysis produces the sum of predicted least-cost paths from each source point to each destination point. The resistant kernel algorithm calculates the cumulative resistance cost-weighted dispersal kernel around each source point up to a user-defined dispersal threshold, providing the rate of organism movement through every pixel in the landscape as a function of the density and number of source points, the dispersal ability of the species, and the resistance of the landscape^38^. The cumulative resistant kernel surface reflects the spatial incidence function of the expected rate of movement of each species through each pixel in the landscape^80,81^.

To account for uncertainties regarding brown bear dispersal abilities^22,40^, we used five distance thresholds in the resistant kernel analyses: 50,000, 100,000, 200,000, 300,000 and 400,000 cost units, which represent movement abilities of 50, 100, 200, 300 and 400 km, respectively, through optimum low resistance habitat. The longest dispersal distances recorded for brown bear are 90 km for a female and 467 km for a male in Norway^82^, therefore, this range of modeled dispersal distances brackets the expected dispersal capability of the species.

We calculated the factorial least-cost path network without a dispersal threshold^77^ to provide a broad-scale assessment of the regional pattern of potential linkage and to map potential long-distance corridors. The buffered least-cost paths were then combined through summation^37^ to produce maps of connectivity among all pairs of presence points.

The resistant kernel connectivity maps were used to identify brown bear core areas^9,15^. We defined core habitat patches as contiguous patches with resistant kernel values > 25% of the highest recorded for the species^29,40^. To evaluate the effectiveness of the current network of protected areas in providing connectivity for this species in Iran, we quantified the extent and percentage of predicted core areas and corridors for this species within the current network.

### Conservation prioritization of core habitats

We prioritized core habitat patches based on probability of connectivity (dPC)^83^ and integral index of connectivity (dIIC)^84^ for all identified core habitats across the five dispersal distance scenarios (i.e. 50, 100, 200, 300 and 400 km) in Conefor 2.6^85^. The dPC and dIIC indices are frequently used as connectivity measures in conservation prioritization studies^42,43,86,87^. The dIIC index considers both habitat amount and habitat reachability across the habitat network, and linkages as dispersal events between patches^84,86^. dIIC also quantifies the loss of connectivity if a patch is removed from the habitat network and can be decomposed into dIICflux (dIICf), dIICconnector (dIICc), dIICintra (dIICi). dPC also considers both habitat amount and habitat reachability, and the probability of dispersal between patches, and can be decomposed into dPCflux (dPCf), dPCconnector (dPCc) and dPCintra (dPCi)^84,86^. dPCintra and dIICintra measure intra-patch connectivity, while flux fraction of a particular node (dPCflux and dIICflux) reflects both patch attributes (e.g., area of suitable habitats) and its position within the landscape, while connector fraction (dIICconnector and dPCconnector) depend only on the topological position of a patch in the landscape^88^. Connector fraction quantifies the importance of the node as a stepping-stone for dispersal, i.e. facilitating dispersal between distant nodes^86^. We used a distance-probability value of 0.5 and 0.05 for minimum and maximum dispersal distances, respectively, as recommended by Saura and Torne^84^.

To quantify differences in the predicted extent and configuration of habitat, we calculated a suite of fragmentation metrics on predicted core habitat patches using FRAGSTATS software^30,45^. To conduct the FRAGSTATS analysis, we first converted the UNICOR resistant kernel outputs into patches by applying a cutoff value^31^. For each species, any values above 25th percentile of the highest dispersal scenario were reclassified as 1, representing habitat patches of high connectivity. Everything else was reclassified as 0. Then, we calculated four class level metrics using FRAGSTATS v4.2.1^45^ including: (1) percentage of the landscape (PLAND), which quantifies the habitat patches of high connectivity as a percentage of the study area; (2) area-weighted mean radius of gyration (GYRATE_AM) or correlation length, which provides a measurement of the extensiveness of habitat patches of high connectivity; (3) largest patch index (LPI), which represents the percentage of the landscape comprised by the largest habitat patch of high connectivity; and (4) number of isolated patches (NP), which provides a measure of the degree of fragmentation. These metrics have been used frequently in past connectivity research^31,41,44,88^, and were shown through simulation to be strong predictors of functional landscape connectivity and gene flow^80^.

## Results

### Multi-scale habitat modeling

Based on the MaxentVariableSelection results, nine variables (with VIF < 3) were chosen to model brown bear habitat in Iran (Supplementary Materials, Tables S1 and S2). These included, in order of variable importance (1) Roughness, (2) Bio1, (3) Bio12, (4) Orchard density, (5) Forest density, (6) Grassland density, (7) NDVI, (8) Distance from rivers, (9) Human footprint. AUC and TSS for all models were > 0.9 and > 0.7, respectively, indicating strong performance of all models run (Table 1). The 16 km scale had the highest AUC and TSS and, therefore, it was chosen for connectivity analysis.

**Table 1:** AUC and TSS of different models for habitat suitability of the brown bear in Iran.

	1 km	2 km	4 km	8 km	16 km	32 km
AUC	0.923	0.935	0.940	0.934	0.954	0.948
TSS	0.836	0.832	0.802	0.727	0.849	0.794

**Figure 2:**
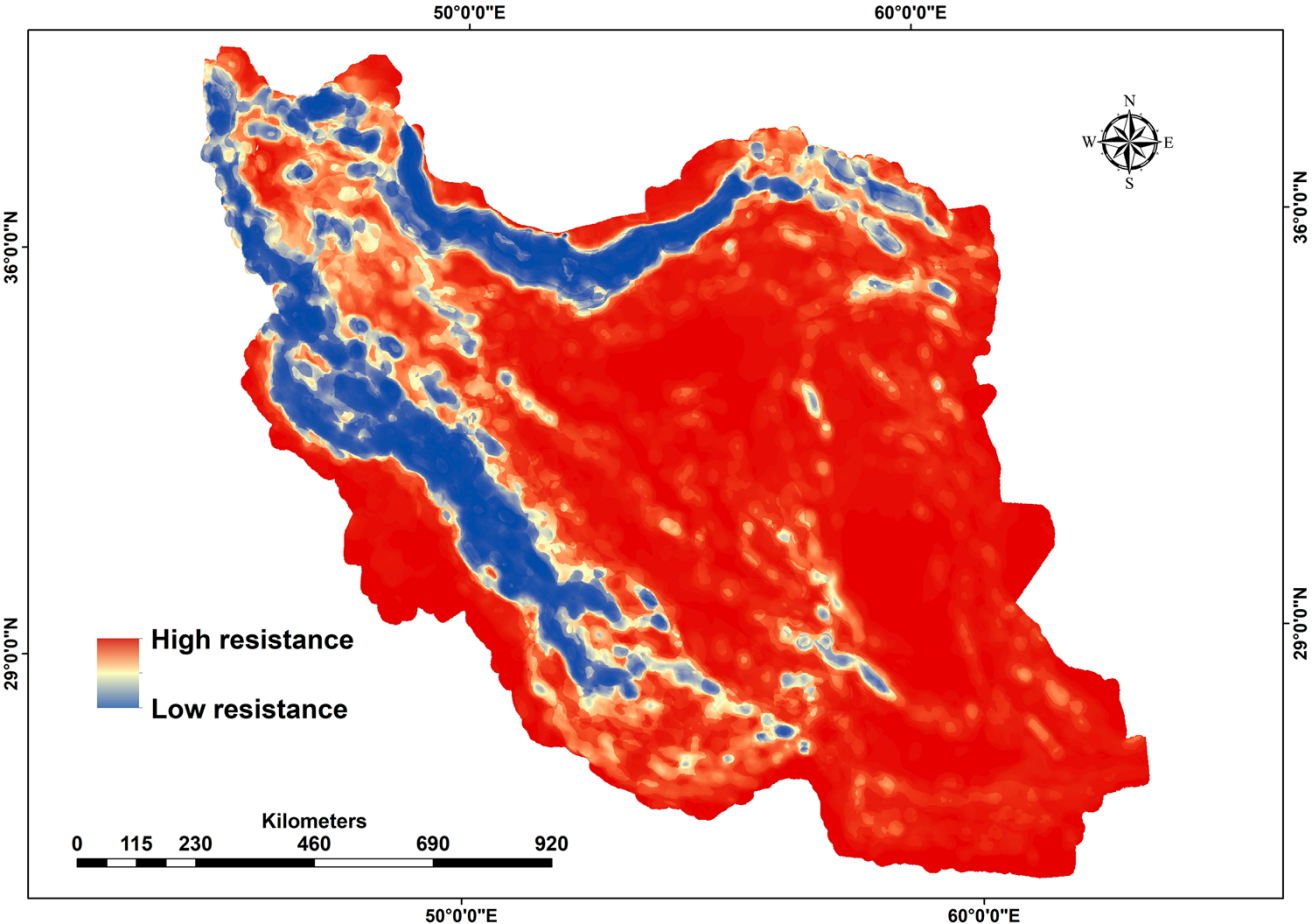
Resistance map of the brown bear at the 16 km scale in Iran.

Based on the 16 km scale model, forest density, roughness, NDVI and human footprint were the most influential variables predicting potential habitat for bears in Iran. Excluding human footprint, all these variables emerged also as the most influential predictors in the other spatial scales considered (Table S3). Roughness, forest density, and NDVI (both natural and artificial vegetation; i.e., forests and orchards) showed a positive relationship with the probability of bear occurrence increased (Fig. S1). Plain areas in the central portion of Iran, along with the northern and southern parts of the country, had the highest resistance, and extensive areas of Zagros and Alborz had the lowest resistance for the brown bear at the scale 16 km (Fig. 2). Other scales showed similar patterns (Fig. S2).

### Brown bear core habitat and connectivity network

**Figure 3:**
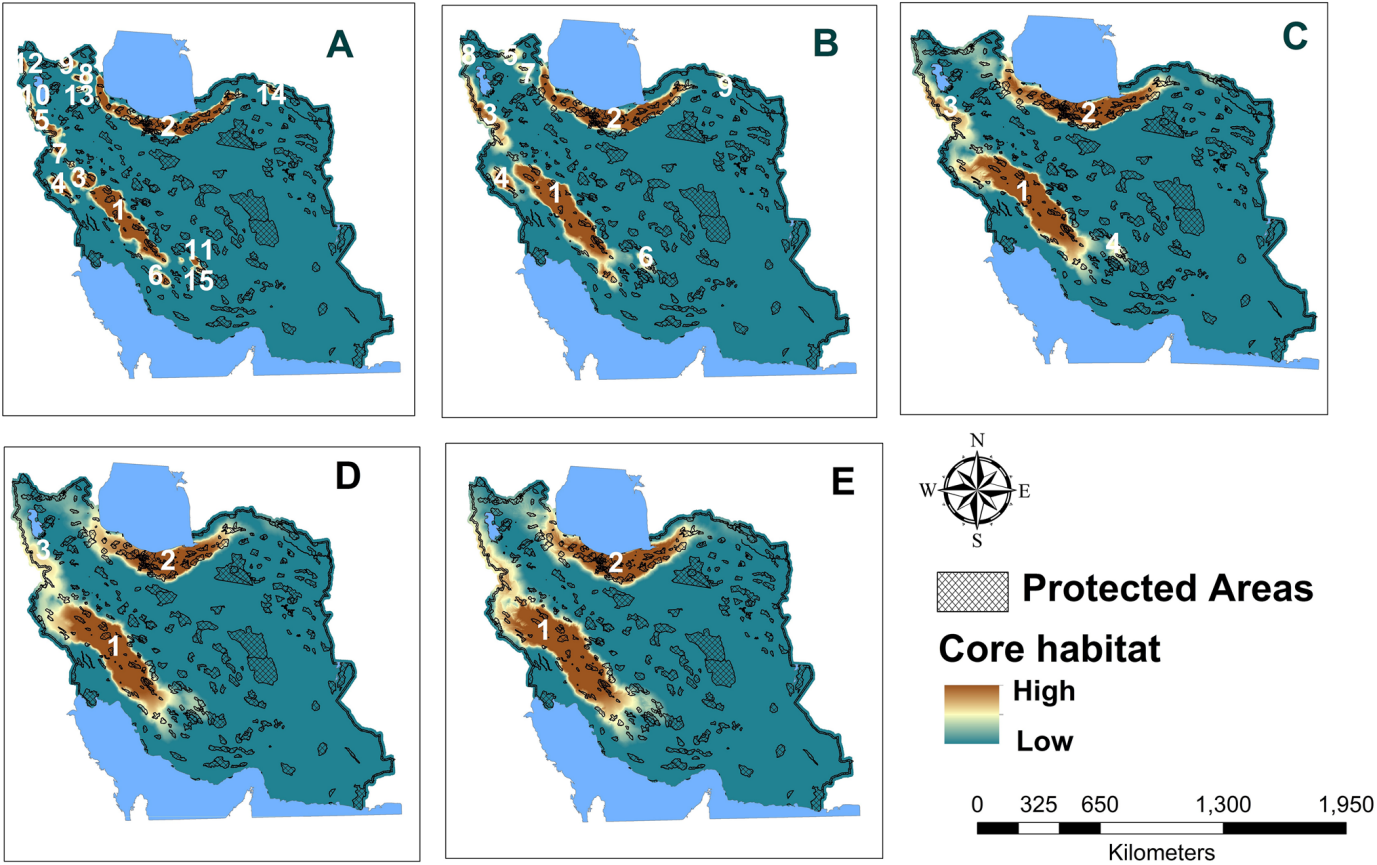
Brown bear core habitats at different dispersal distances: 50 (**A**), 100 (**B**), 200 (**C**), 300 (**D**) and 400 (**E**) km, and network of Iranian protected areas. Mean values of dPC used for prioritizing core habitats at five dispersal scenarios are shown.

Our connectivity simulation modeling for the brown bear revealed that most core habitat and connectivity areas are concentrated in the northern and northeastern (Alborz Mountains) and northwestern to southwestern parts of the study area (Zagros Mountains) (Fig. 3). Overall, we identified 15 core areas (107,233.67 km^2^) at a dispersal distance of 50,000 cost units (corresponding to limit of female dispersal ability), of which two were particularly larger than 30,000 km^2^ (Core 1 and 2) with a total area of 75,911.57 km^2^ located in Zagros and Alborz Mountains. Thirty-one percent (31%) of Core 2 is covered by protected areas. In contrast, only 13.8% of Core 1 is covered by protected areas.

Depending on assumed brown bear dispersal distances, between 17.77 and 22.10% of predicted bear core areas overlap with protected areas (Table 2). Also, the density of roads inside predicted core habitats for the same dispersal distance of 50 km is 239.63 m/km^2^ (Table 2). Overall, 12 No Hunting Areas, 17 Protected Areas and 1 National Park overlap with brown bear core habitats in the Zagros Mountains (Cores 1, 3, 4, 5, 6, 7, 10, 11, 12 and 15) and 17 No Hunting Areas, 14 Protected Areas, 1 Wildlife refuge and 2 National Park covers brown bear core habitats in Alborz Mountains (Core 2, 8, 13 and 14) (Table S4).

**Table 2:** Extent and percentage of brown bear core habitats within current conservation networks in Iran based on different estimated bear dispersal distances.

Estimated dispersal distance (km)	Extent of core habitats (km^2^)	Extent of protected core habitats (km^2^)	Percentage of protected core habitats	Road density (m/km^2^)
50	155,494.69	34,371.47	22.10	239.63
100	163,582.76	40,167.10	19.54	57.92
200	187,145.24	31,379.51	19.18	65.19
300	205,474.68	34,124.61	18.23	67.75
400	233,736.78	41,542.29	17.77	69.60

**Figure 4:**
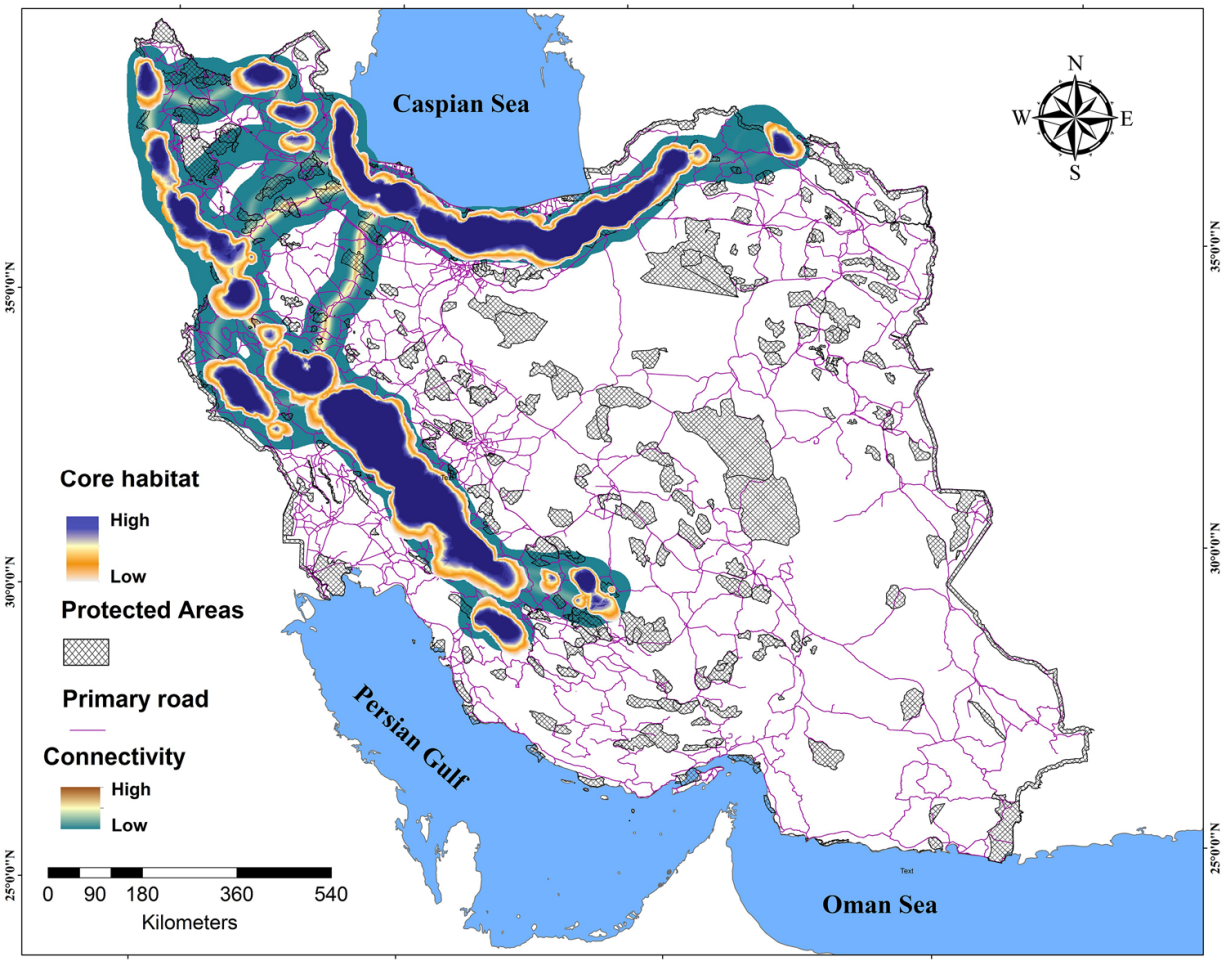
The estimated suitability of core habitats for brown bear in Iran and the corridors strength connecting them from weak(green) to strong (brown) at dispersal ability 50 km in Iran.

The strongest predicted connectivity for brown bear was between the northern and western bear population core areas, specifically between core areas 1, 2, 3 and 7 (Fig. 3). Based on our calculation, approximately 21% of the entire landscape was favorable to brown bear movements, but with varying degrees of strength. For this species, the lowest degree of connectivity was estimated for northeastern, northwestern and southeastern parts of the brown bear Iran distribution (Fig. 4). Based on the density of least-cost paths, the southern and northern nuclei were predicted to be the most isolated. The 29.43% of this corridor network falls within protected areas, but most predicted corridor paths are bisected multiple times by roads (Fig. 4 and Table 3).

**Table 3:** The extent and percent of corridors covered by current conservation networks for brown bear in Iran.

Extent of corridors (km^2^)	Extent of protected corridors (km^2^)	% of protected corridors	Length of paved roads crossing the corridor path (km)	Road density (m/km^2^)
15,866.39	4670.50	29.43	1236.87	264.82

### Identification of top-ranked brown bear core habitats

The least important predicted habitat core changed with simulated dispersal ability. For example, at a dispersal distance of 50 km, Cores 13, 14 and 15 had the smallest contribution to overall habitat connectivity, while for distances of 100 km Cores 14, 15 and 12 were replaced with Cores 9, 6 and 8. This result revealed that with increasing dispersal distance, there were important changes in the relative importance of different patches and an overall increase in connectivity importance across patches. Based on dPCc, Core 3 was the most important stepping stone among other patches at dispersal ability 50–300 km. At dispersal ability of 50 and 100 km, Core 1 and 5 had the next most important contributions as stepping stones. Core 2 had large contribution as a stepping stone at dispersal ability 100 km only. Over dispersal distances of 300 and 400 km, there was a decreasing trend in importance of core patches as stepping stones in the connectivity network of the brown bear. Based on dIICc, Core 1 and 2 were the most important patches at dispersal distance 50–200 km.

**Figure 5:**
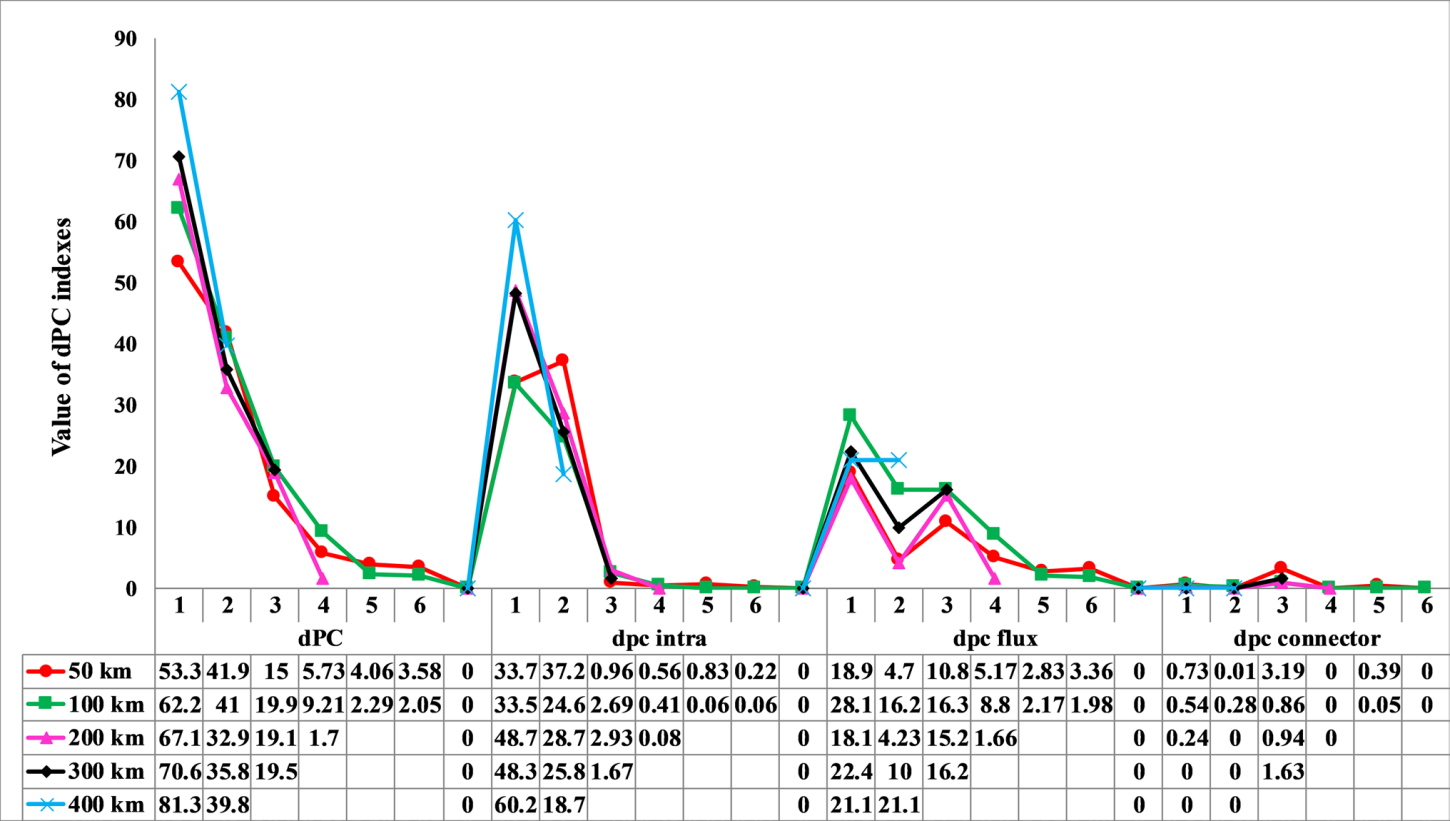
Mean values of dPC index and its three fractions (intra, flux and connector) calculated for predicted 6 top ranked core habitats at five dispersal scenarios (50, 100, 200, 300 and 400 km).

**Figure 6:**
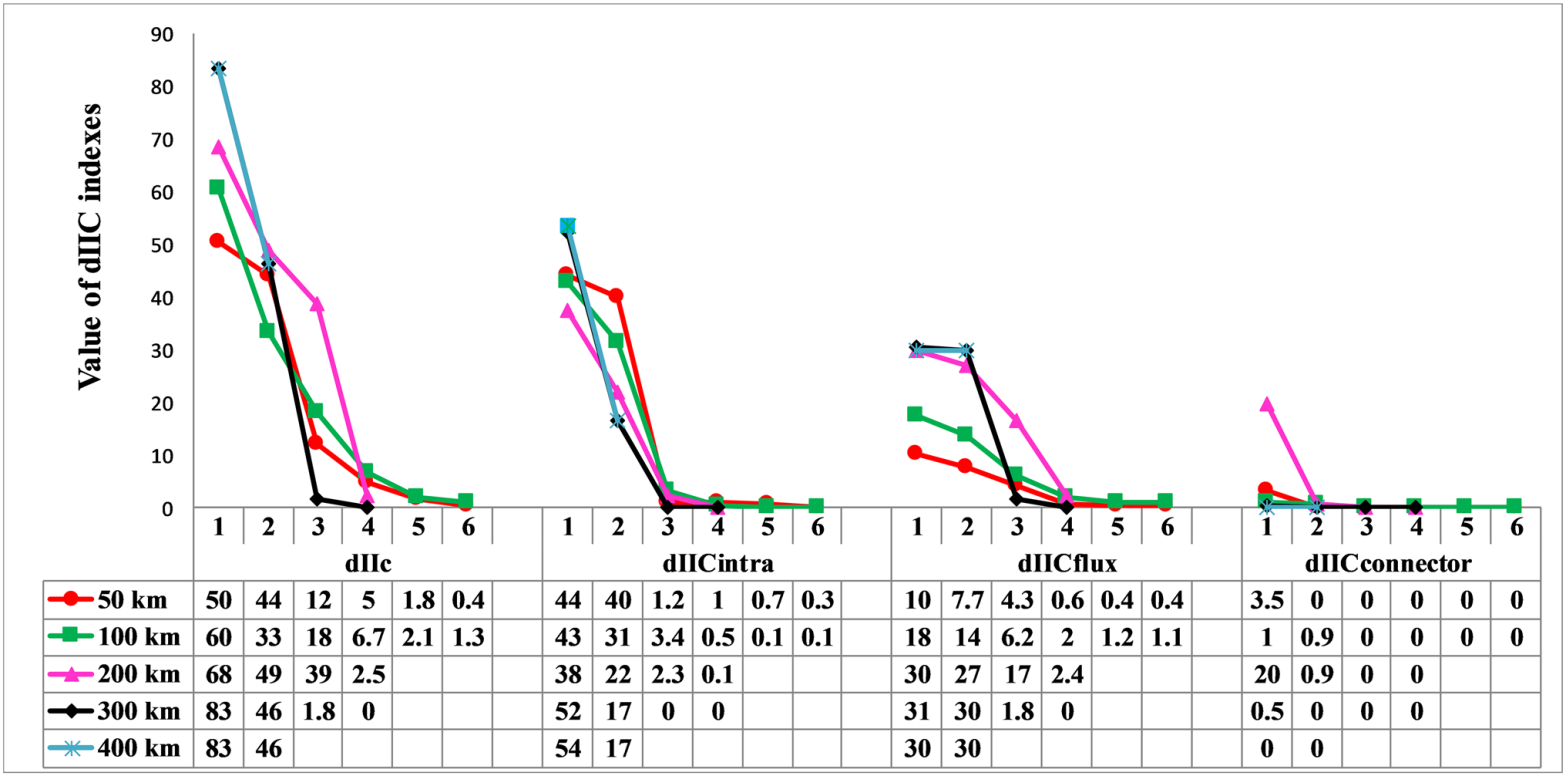
Mean values of dIIC index and its three fractions (dIICintra, dIICflux and dIICconnector) calculated for 6 top ranked core habitats at five dispersal scenarios (50, 100, 200, 300 and 400 km).

The contribution of core habitats to landscape connectivity revealed a different pattern of ranking according to the dPC index and dispersal distance scenarios (Fig. 5). From the 50 km dispersal distance to 400 km, consistent habitat patches were identified by dIIC and dPC (Figs. 5 and 6). Based on the dPC and dIIC index, the patches 1–6 were the most important patches for maintaining habitat connectivity at dispersal distance 50 and 100 km. Also, the patches 1–4, 1–3 and 1, 2 were the most important patches at dispersal distance 200, 300 and 400 km, respectively (Figs. 5 and 6). Patch 2 was the most important for the dPCi index at 50 km dispersal distance, but from 400 km both dPCi and dIICi identified Patch 1 as the most important (Figs. 5 and 6). Based on dPCf, from 50 to 300 km core 3 was more important than core 2, but this pattern was not observed in dIICf (Figs. 5 and 6). From 50 to 300 km, values of dPCc for Core 3 were higher than Core 1 and 2, but this trend was different for dIICc.

The number of isolated patches decreases with higher dispersal ability, whereas LPI, PLAND and CL rise with higher dispersal ability (Table 4). For this species, 9–13.5% of the landscape is occupied by connected habitat patches depending on dispersal ability.

**Table 4:** FRAGSTATS results for the brown bear. The metrics include: number of individual core patches (NP) largest patch index (LPI), percentage of landscape in connected habitat (PLAND) and correlation length of core habitats (CL) for the brown bear in five levels of dispersal ability (50,000, 100,000, 200,000, 300,000, 400,000).

Dispersal ability (km)	NP	LPI	PLAND	CL
50	15	3.22	9.01	44,031.71
100	10	4.59	9.21	64,490.31
200	3	4.97	9.48	129,175.97
300	3	5.94	10.85	129,606.28
400	2	8.59	13.55	204,022.72

The core habitats were defined as contiguous units with resistant kernel values > 25% of the highest resistance kernel for the species.

## Discussion

Our results highlight that crucial core habitats for brown bear conservation in Iran are concentrated in the Zagros (core 1) and Alborz (core 2) Mountains with important corridors between them. These two chains of mountains have a high level of biological diversity and endemism^58,89^ which makes them two of the most valuable landscapes across the country from a conservation perspective. Local-scale studies revealed low connectivity among patches of brown bear habitat^90^. However, Ashrafzadeh et al.^91^ applied circuit theory^36^ to model connectivity across the brown bear distribution in Iran and inferred strong connectivity between brown bear core habitats despite clear restrictions such as anthropogenic activity. This is because the circuit theory approach did not incorporate scale dependency of dispersal ability, which has been shown to dominate predictions of connectivity^17,22,80,92^.

One of the main strengths of the resistant kernel approach as an alternative connectivity modeling method is its explicit and realistic incorporation of dispersal thresholds^93^. The dispersal ability of a species can affect core habitats and connectivity predictions significantly. Our findings are in accordance with previous studies^22,29,42–44^. Indeed, several past research studies have shown that often dispersal ability is much more influential on connectivity predictions than relative landscape resistance or connectivity algorithm^22,80,92^. Therefore, based on our results, we believe that previous predictions likely overestimate connectivity of brown bear in Iran. In particular, our modeling shows that dispersal limitations that likely pertain to female brown bears result in highly disjointed and fragmented populations. Critically, dispersal of females is required for a population to colonize and reestablish a breeding population in new parts of its former range.

Another important difference between our results and those of Ashrafzadeh et al.^91^ is that they reported substantial landscape resistance amongst Alborz and Zagros, and Alborz and Arasbaran^91^, which is inconsistent with the results of our analyses. Our analysis suggests that at realistic dispersal abilities these two main core areas are internally well connected.

These two core habitat parts for the brown bear population (Zagros and Alborz) were predicted to be broken up into a total of fifteen isolated patches if dispersal of brown bear is limited to 50 km (approximately the limit of female dispersal), but with a dispersal ability of 200– 400 km (corresponding to the limit of male dispersal) it would result in three to two habitat patches. Our findings implied high sensitivity of the extent and fragmentation of connected habitat as a function of dispersal ability, and also to highly divergent connectivity for male vs female bears. Therefore, considerable effort should be invested in improving understanding of dispersal behavior and functional connectivity of large carnivores such as brown bear to increase the precision of core area delineation and prioritization of corridor paths in Iran.

Our factorial least-cost path analysis identified optimal routes between these areas in order to facilitate connectivity. This spatially-explicit information could guide conservation practitioners to implement landscape conservation strategies. Similar work using these methods in southern Africa^9,22^ and Southeast Asia^15,16,18^ applied scenario optimization strategies to evaluate the relative impacts of alternative conservation and development scenarios. Our results provide a strong base for future scenario modeling of this kind in Iran. The information provided here, however, in itself is useful in identifying and prioritizing the most important core areas and corridors for brown bear^9^ and identifying where they are most threatened by land use and roads^40^.

Based on both AUC and TSS indices, the best habitat suitability model was the one at the 16 km scale, suggesting, as previously seen for this species in Spain^25^, that brown bear habitat selection is dominated by broad-scales of environmental variation. Brown bears prefer high density of forests, areas of high topographical roughness, high vegetation density and low human footprint in Iran. This is consistent with other studies of the species across its range^25,94,95^. Similar predictors have been highlighted for bears in other areas^25,90,92,96–99^.

Proper design of protected areas and protected area networks should incorporate spatially explicit prioritization of both core areas and connectivity networks among them, which will ensure that protected areas functionally protect focal populations and enable dispersal among them, which facilitates demographic rescue and gene flow which are crucial for long-term species conservation^21,100^. In our case, the intersection of existing Iranian protected areas with our identified core habitats was relatively low. Only 29.43% of the predicted core area network of brown bear overlapped with protected areas. Therefore, our results can guide to conservation practitioners to establish new protected areas in strategic locations most important within the full conservation network^101^. Protecting a habitat large enough to sustain the life history traits of brown bear as an umbrella species will likely also protect the long-term persistence of many other species^99,102^.

Among the identified core habitats, the highest overlap between core habitats and protected areas was observed for core 1 and core 2. These two core habitats have also been documented to have a high potential for supporting other large carnivores of conservation concern, such as Persian leopards (*Panthera pardus saxicolor*)^86^. However, the coverage of protected areas could be improved for other identified core habitats which our analysis shows are relatively unprotected. Similarly, Moqanaki and Cushman^41^ and Khosravi et al.^42^, predicted that the distribution of protected areas in Iran was an important factor determining the occurrence and dispersal of Asiatic cheetah and sympatric carnivores.

The strategic expansion of protected areas to provide stepping stones and augmentation to the key core areas should be accompanied by the establishment of conservation actions in the linkages between protected core areas to increase functional landscape connectivity for carnivores^9^. The most robust functional corridors for the brown bear were predicted between core 1, 2, 3 and 7 (Fig. 5). We recommend prioritization of establishing new protected areas along these key connectivity routes, land use zonation and management actions along these routes to reduce mortality risk and human-wildlife conflict.

Most protected area networks in developing countries such as Iran are fragmented by roads, and road collisions are a serious threat for carnivores^41,42^. In addition to reducing dispersal success, roads also can cause high rates of direct mortality (wildlife vehicle collisions) which can be serious threat for brown bears^103^. This problem will be aggravated when roads bisect corridors^104–106^. Our results identified vulnerable parts of the connectivity network in Core 1 and 2 where roads intersected strong movement corridors^34,40^. Vulnerability of these locations is related to the potential for brown bear vehicle collisions, and concentration of human activities and access in these areas increasing risk of poaching, a common threat for bears in human-modified landscapes^98,100^. Our findings in this regard are similar to those of Moqanaki and Cushman^41^ and Khosravi et al.^42^, who both identified multiple instances where primary and secondary roads cross the predicted corridor paths between these core patches. Our results should be combined with those of Moqanaki and Cushman^41^ and Khosravi et al.^42^ to prioritize road segments for mitigating mortality risk and improving connectivity, such as through fencing and overpass structures to funnel dispersing animals across the road in optimal locations for network connectivity.

Given uncertainty in the functional dispersal distances of existing brown bear populations, plus the high sensitivity of populations to this parameter, it is important to evaluate a range of dispersal distance scenarios, identify dispersal-scale thresholds, and develop multi-scale conservation recommendations. That is one of the strengths of the approach we presented in this paper. By evaluating connectivity and prioritizing core areas and corridors across a range of dispersal distances, we identify the key nodes and linkages across the brown bear population in Iran and quantify its sensitivity to a scale of dispersal behavior. The dispersal distances we modeled bracket the expected functional responses of brown bears. Female brown bears are known to be more risk averse, more philopatric and less mobile than males. The lower end of our dispersal distance simulation suggests that female brown bears are unlikely to disperse distances necessary to recolonize most extant habitat patches. Therefore, for population recovery and range recolonization relocation of female brown bears to areas where male brown bears are known to disperse to may be an important conservation strategy.

When designating vast protected areas is politically intractable^107^, an alternative may be to develop networks of interconnected protected areas. Managing and maintaining functional corridors may be more feasible in some contexts than establishing new protected areas^108^. These connected protected area networks could aid gene flow^21^ and prevent isolation of small populations^92^. Due to tendency of bears to avoid humans, connecting habitats for their survival is crucial^27^. However, we also urge caution in the tempting idea that maintaining connectivity can mitigate for habitat loss. In most cases, it cannot. Therefore, carnivore conservation should focus on maintaining core habitat quality and extent as the primary focus and then establishing corridor networks linking protected core areas, with conflict management and efforts to foster human-wildlife coexistence across the landscape.

It should also be noted that findings of connectivity studies based on presence points and habitat models are limited by uncertainties^32,33^. Specifically, our analysis uses a relatively modest sample of presence-only data, which could limit the power of our predictions. However, the high performance of our models using bootstrapped and cross-validated model assessment suggests that our habitat predictions are robust. Habitat quality, however, is not the same as connectivity^32,33,75^. It would be better to fit connectivity models with movement^76^ or gene flow^108^ data instead of habitat models, as dispersal is often related to different factors at different scales than home range habitat selection. Therefore, satellite tracking^76,88^ and landscape genetic^31,108^ studies are also necessary to make more reliable predictions and validate findings carried out from connectivity prioritizations made on the basis of habitat selection. To conclude, habitat suitability and connectivity models should be considered the first step towards building a nation-wide strategy for corridor improvement^101^. Optimizing habitat protection and connectivity^15,18^ should be a core component of efforts to plan the establishment of new protected areas, particularly for large, vulnerable and highly mobile species such as carnivores. Furthermore, to facilitate human-brown bear coexistence outside protected areas DoE should consider implementing some approaches such as reduction of human-induced mortality, safeguard habitat connectivity; mitigate road effects^109^ and educate local communities.

## Supplementary Information

Supplementary Information.

## References

[1] Kopatz A (2012). Connectivity and population subdivision at the fringe of a large brown bear (*Ursus arctos*) population in North Western Europe. Conserv. Genet..

[2] Mohammadi A, Kaboli M (2016). Evaluating wildlife–vehicle collision hotspots using kernel-based estimation: a focus on the endangered Asiatic cheetah in central Iran. Hum. Wildl. Interact..

[3] Murphy SM (2017). Consequences of severe habitat fragmentation on density, genetics, and spatial capture–recapture analysis of a small bear population. PLoS ONE.

[4] Hosseini-Zavarei F, Farhadinia MS, Beheshti-Zavareh M, Abdoli A (2013). Predation by grey wolf on wild ungulates and livestock in central Iran. J. Zool..

[5] Tumendemberel O (2019). Phylogeography, genetic diversity, and connectivity of brown bear populations in Central Asia. PLoS ONE.

[6] Hilty JA, Lidicker WZ, Merenlender AM (2012). Corridor Ecology: The Science and Practice of Linking Landscapes for Biodiversity Conservation.

[7] Cushman SA (2011). Limiting factors and landscape connectivity: the American marten in the Rocky Mountains. Landsc. Ecol..

[8] Oriol-Cotterill A, Valeix M, Frank LG, Riginos C, Macdonald DW (2015). Landscapes of coexistence for terrestrial carnivores: the ecological consequences of being downgraded from ultimate to penultimate predator by humans. Oikos.

[9] Cushman SA (2018). Prioritizing core areas, corridors and conflict hotspots for lion conservation in southern Africa. PLoS ONE.

[10] Rio-Maior H, Nakamura M, Álvares F, Beja P (2019). Designing the landscape of coexistence: integrating risk avoidance, habitat selection and functional connectivity to inform large carnivore conservation. Biol. Conserv..

[11] Macdonald DW (2019). Multi-scale habitat modelling identifies spatial conservation priorities for mainland clouded leopards (*Neofelis nebulosa*). Divers. Distrib..

[12] Johansson Ö (2016). Land sharing is essential for snow leopard conservation. Biol. Conserv..

[13] López-Bao JV, Bruskotter J, Chapron G (2017). Finding space for large carnivores. Nat. Ecol. Evol..

[14] Crespin SJ, Simonetti JA (2019). Reconciling farming and wild nature: Integrating human–wildlife coexistence into the land-sharing and land-sparing framework. Ambio.

[15] Kaszta Ż, Cushman SA, Macdonald DW (2020). Prioritizing habitat core areas and corridors for a large carnivore across its range. Anim. Conserv..

[16] Kaszta Ż (2020). Simulating the impact of Belt and Road initiative and other major developments in Myanmar on an ambassador felid, the clouded leopard, *Neofelis nebulosa*. Landsc. Ecol..

[17] Cushman SA, Compton BW, McGarigal K, Cushman SA, Huettmann F (2010). Habitat fragmentation effects depend on complex interactions between population size and dispersal ability: modeling influences of roads, agriculture and residential development across a range of life-history characteristics. Spatial Complexity, Informatics, and Wildlife Conservation.

[18] Kaszta Ż (2019). Integrating Sunda clouded leopard (*Neofelis diardi*) conservation into development and restoration planning in Sabah (Borneo). Biol. Conserv..

[19] Beier P, Majka DR, Spencer WD (2008). Forks in the road: choices in procedures for designing wildland linkages. Conserv. Biol..

[20] Romportl D (2013). Designing migration corridors for large mammals in the Czech Republic. J. Landsc. Ecol..

[21] Ruiz-González A (2014). Landscape genetics for the empirical assessment of resistance surfaces: the European pine marten (*Martes martes*) as a target-species of a regional ecological network. PLoS ONE.

[22] Cushman SA, Elliot NB, Macdonald DW, Loveridge AJ (2016). A multi-scale assessment of population connectivity in African lions (*Panthera leo*) in response to landscape change. Landsc. Ecol..

[23] Linnell, J., Salvatori, V. & Boitani, L. Guidelines for population level management plans for large carnivores in Europe. A Large Carnivore Initiative for Europe (2008).

[24] Reljic S (2018). Challenges for transboundary management of a European brown bear population. Glob. Ecol. Conserv..

[25] Mateo Sanchez MC, Cushman SA, Saura S (2014). Scale dependence in habitat selection: the case of the endangered brown bear (*Ursus arctos*) in the Cantabrian Range (NW Spain). Int. J. Geogr. Inf. Sci..

[26] Vergara M, Cushman SA, Urra F, Ruiz-González A (2016). Shaken but not stirred: multiscale habitat suitability modeling of sympatric marten species (*Martes martes* and *Martes foina*) in the northern Iberian Peninsula. Landsc. Ecol..

[27] Ziółkowska E (2016). Assessing differences in connectivity based on habitat versus movement models for brown bears in the Carpathians. Landsc. Ecol..

[28] Sarkar MS (2018). Multiscale statistical approach to assess habitat suitability and connectivity of common leopard (*Panthera pardus*) in Kailash Sacred Landscape, India. Spat. Stat..

[29] Ashrafzadeh MR (2020). A multi-scale, multi-species approach for assessing effectiveness of habitat and connectivity conservation for endangered felids. Biol. Conserv..

[30] McGarigal K, Wan HY, Zeller KA, Timm BC, Cushman SA (2016). Multi-scale habitat selection modeling: a review and outlook. Landsc. Ecol..

[31] Wasserman TN, Cushman SA, Shirk AS, Landguth EL, Littell JS (2012). Simulating the effects of climate change on population connectivity of American marten (*Martes americana*) in the northern Rocky Mountains, USA. Landsc. Ecol..

[32] Mateo-Sánchez MC (2015). A comparative framework to infer landscape effects on population genetic structure: Are habitat suitability models effective in explaining gene flow?. Landsc. Ecol..

[33] Zeller KA (2018). Are all data types and connectivity models created equal? Validating common connectivity approaches with dispersal data. Divers. Distrib..

[34] Cushman SA, Lewis JS, Landguth EL (2014). Why did the bear cross the road? Comparing the performance of multiple resistance surfaces and connectivity modeling methods. Diversity.

[35] Adriaensen F (2003). The application of ‘least-cost’modelling as a functional landscape model. Landsc. Urban Plan..

[36] McRae BH (2006). Isolation by resistance. Evolution (N. Y.).

[37] Cushman SA, McKelvey KS, Schwartz MK (2009). Use of empirically derived source–destination models to map regional conservation corridors. Conserv. Biol..

[38] Compton BW, McGarigal K, Cushman SA, Gamble LR (2007). A resistant-kernel model of connectivity for amphibians that breed in vernal pools. Conserv. Biol..

[39] Panzacchi M (2016). Predicting the continuum between corridors and barriers to animal movements using step selection functions and randomized shortest paths. J. Anim. Ecol..

[40] Cushman SA, Lewis JS, Landguth EL (2013). Evaluating the intersection of a regional wildlife connectivity network with highways. Mov. Ecol..

[41] Moqanaki EM, Cushman SA (2017). All roads lead to Iran: predicting landscape connectivity of the last stronghold for the critically endangered Asiatic cheetah. Anim. Conserv..

[42] Khosravi R, Hemami M, Cushman SA (2018). Multispecies assessment of core areas and connectivity of desert carnivores in central Iran. Divers. Distrib..

[43] Shahnaseri G (2019). Contrasting use of habitat, landscape elements, and corridors by grey wolf and golden jackal in central Iran. Landsc. Ecol..

[44] Cushman, S. A. & Landguth, E. L. Ecological associations, dispersal ability, and landscape connectivity in the northern Rocky Mountains. In *Research Paper RMRS-RP-90. Fort Collins, CO: U.S. Department of Agriculture, Forest Service, Rocky Mountain Research Station*. vol. 90, 21 p (2012).

[45] McGarigal K, Cushman SA (2002). Comparative evaluation of experimental approaches to the study of habitat fragmentation effects. Ecol. Appl..

[46] Cozzi G (2016). Anthropogenic food resources foster the coexistence of distinct life history strategies: year-round sedentary and migratory brown bears. J. Zool..

[47] McLellan, B. N., Proctor, M. F., Huber, D. & Michel, S. Ursus arctos (amended version of 2017 assessment). The IUCN Red List of Threatened Species 2017: e. T41688A121229971 (2017).

[48] Penteriani V, Melletti M (2020). Bears of the World: Ecology, Conservation and Management.

[49] Wolf C, Ripple WJ (2017). Range contractions of the world’s large carnivores. R. Soc. Open Sci..

[50] Garshelis D, McLellan B (2011). Are bear subspecies a thing of the past?. Int. Bear News.

[51] Hajjar I (2011). The Syrian bear still lives in Syria. Int. Bear News.

[52] Calvignac S, Hughes S, Hänni C (2009). Genetic diversity of endangered brown bear (*Ursus arctos*) populations at the crossroads of Europe, Asia and Africa. Divers. Distrib..

[53] Ansari M, Ghoddousi A (2018). Water availability limits brown bear distribution at the southern edge of its global range. Ursus.

[54] Ashrafzadeh MR, Kaboli M, Naghavi MR (2016). Mitochondrial DNA analysis of Iranian brown bears (*Ursus arctos*) reveals new phylogeographic lineage. Mamm. Biol..

[55] Gutleb B, Ziaie H (1999). On the distribution and status of the Brown Bear, *Ursus arctos*, and the Asiatic Black Bear, *U. thibetanus*, Iran. Zool. Middle East.

[56] Moqanaki EM, Jiménez J, Bensch S, López-Bao JV (2018). Counting bears in the Iranian Caucasus: remarkable mismatch between scientifically-sound population estimates and perceptions. Biol. Conserv..

[57] Yusefi GH, Faizolahi K, Darvish J, Safi K, Brito JC (2019). The species diversity, distribution, and conservation status of the terrestrial mammals of Iran. J. Mammal..

[58] Almasieh K, Rouhi H, Kaboodvandpour S (2019). Habitat suitability and connectivity for the brown bear (*Ursus arctos*) along the Iran–Iraq border. Eur. J. Wildl. Res..

[59] Nezami B, Farhadinia MS (2011). Litter sizes of brown bears in the Central Alborz Protected Area, Iran. Ursus.

[60] Darvishsefat, A. A. *Atlas of Protected Areas of Iran*. (Ravi, 2006).

[61] Atzeni L (2020). Meta-replication, sampling bias, and multi-scale model selection: a case study on snow leopard (*Panthera uncia*) in western China. Ecol. Evol..

[62] Ambarli, H., Erturk, A. & Soyumert, A. Current status, distribution, and conservation of brown bear (Ursidae) and wild canids (gray wolf, golden jackal, and red fox; Canidae) in Turkey (2016).

[63] Brown JL (2014). SDM toolbox: a python-based GIS toolkit for landscape genetic, biogeographic and species distribution model analyses. Methods Ecol. Evol..

[64] Evans, J. S. & Oakleaf, J. Geomorphometry and gradient metrics toolbox (ArcGIS 10.0) (2012).

[65] Ghorbanian A (2020). Improved land cover map of Iran using Sentinel imagery within Google Earth Engine and a novel automatic workflow for land cover classification using migrated training samples. ISPRS J. Photogram. Remote Sens..

[66] Sanderson EW (2002). The human footprint and the last of the wild: the human footprint is a global map of human influence on the land surface, which suggests that human beings are stewards of nature, whether we like it or not. Bioscience.

[67] Fick SE, Hijmans RJ (2017). WorldClim 2: new 1-km spatial resolution climate surfaces for global land areas. Int. J. Climatol..

[68] Jueterbock, A. ‘MaxentVariableSelection’vignette. (2015).

[69] R Development Core, team. A Language ans Environment for Statistical Computing. *R Found Stat. Comput. Vienna Austria* **2**, (2018).

[70] Naimi B, Hamm NAS, Groen TA, Skidmore AK, Toxopeus AG (2014). Where is positional uncertainty a problem for species distribution modelling?. Ecography (Cop.).

[71] Zuur AF, Ieno EN, Elphick CS (2010). A protocol for data exploration to avoid common statistical problems. Methods Ecol. Evol..

[72] Liaw A, Wiener M (2002). Classification and regression by randomForest. R News.

[73] Evans JS, Cushman SA (2009). Gradient modeling of conifer species using random forests. Landsc. Ecol..

[74] Wasserman TN, Cushman SA, Schwartz MK, Wallin DO (2010). Spatial scaling and multi-model inference in landscape genetics: Martes Americana in Northern Idaho. Landsc. Ecol..

[75] Cushman SA, Lewis JS (2010). Movement behavior explains genetic differentiation in American black bears. Landsc. Ecol..

[76] Cushman SA, Macdonald EA, Landguth EL, Malhi Y, Macdonald DW (2017). Multiple-scale prediction of forest loss risk across Borneo. Landsc. Ecol..

[77] Zeller KA, McGarigal K, Whiteley AR (2012). Estimating landscape resistance to movement: a review. Landsc. Ecol..

[78] Wan HY, Cushman SA, Ganey JL (2019). Improving habitat and connectivity model predictions with multi-scale resource selection functions from two geographic areas. Landsc. Ecol..

[79] Landguth EL, Hand BK, Glassy J, Cushman SA, Sawaya MA (2012). UNICOR: a species connectivity and corridor network simulator. Ecography (Cop.).

[80] Cushman SA, Landguth EL, Flather CH (2013). Evaluating population connectivity for species of conservation concern in the American Great Plains. Biodivers. Conserv..

[81] Kaszta Ż, Cushman SA, Sillero-Zubiri C, Wolff E, Marino J (2018). Where buffalo and cattle meet: modelling interspecific contact risk using cumulative resistant kernels. Ecography (Cop.).

[82] Støen, O.-G. *Natal Dispersal and Social Organization in Brown Bears*. (Norwegian University of Life Sciences, Department of Ecology and Natural, 2006).

[83] Saura S, Pascual-Hortal L (2007). A new habitat availability index to integrate connectivity in landscape conservation planning: comparison with existing indices and application to a case study. Landsc. Urban Plan..

[84] Saura S, Torné J (2009). Conefor Sensinode 2.2: a software package for quantifying the importance of habitat patches for landscape connectivity. Environ. Model. Softw..

[85] Avon C, Bergès L (2016). Prioritization of habitat patches for landscape connectivity conservation differs between least-cost and resistance distances. Landsc. Ecol..

[86] Farhadinia M (2020). SPECIES OR SPACE: a combined gap analysis to guide management planning of conservation areas. Landsc. Ecol..

[87] Saura S, Rubio L (2010). A common currency for the different ways in which patches and links can contribute to habitat availability and connectivity in the landscape. Ecography (Cop.).

[88] Elliot NB, Cushman SA, Macdonald DW, Loveridge AJ (2014). The devil is in the dispersers: predictions of landscape connectivity change with demography. J. Appl. Ecol..

[89] Noroozi J, Akhani H, Breckle S-W (2008). Biodiversity and phytogeography of the alpine flora of Iran. Biodivers. Conserv..

[90] Habibzadeh N, Ashrafzadeh MR (2018). Habitat suitability and connectivity for an endangered brown bear population in the Iranian Caucasus. Wildl. Res..

[91] Ashrafzadeh M-R, Khosravi R, Ahmadi M, Kaboli M (2018). Landscape heterogeneity and ecological niche isolation shape the distribution of spatial genetic variation in Iranian brown bears, *Ursus arctos* (Carnivora: Ursidae). Mamm. Biol..

[92] Ash E, Cushman SA, Macdonald DW, Redford T, Kaszta Ż (2020). How important are resistance, dispersal ability, population density and mortality in temporally dynamic simulations of population connectivity? A case study of tigers in southeast Asia. Land.

[93] Cushman SA, Macdonald DW, Willis KJ (2013). Biological corridors and connectivity [Chapter 21]. Key Topics in Conservation Biology 2.

[94] Ghoddousi, A. Habitat suitability modelling of the Brown bear *Ursus arctos* in Croatia and Slovenia using telemetry data (2010).

[95] Steyaert SMJG (2016). Ecological implications from spatial patterns in human-caused brown bear mortality. Wildl. Biol..

[96] Güthlin D (2011). Estimating habitat suitability and potential population size for brown bears in the Eastern Alps. Biol. Conserv..

[97] Penteriani V (2018). Evolutionary and ecological traps for brown bears *Ursus arctos* in human-modified landscapes. Mamm. Rev..

[98] Zarzo-Arias A (2019). Identifying potential areas of expansion for the endangered brown bear (*Ursus arctos*) population in the Cantabrian Mountains (NW Spain). PLoS ONE.

[99] Morales-González A, Ruiz-Villar H, Ordiz A, Penteriani V (2020). Large carnivores living alongside humans: brown bears in human-modified landscapes. Glob. Ecol. Conserv..

[100] Fedorca A (2019). Inferring fine-scale spatial structure of the brown bear (*Ursus arctos*) population in the Carpathians prior to infrastructure development. Sci. Rep..

[101] Liu C, Newell G, White M, Bennett AF (2018). Identifying wildlife corridors for the restoration of regional habitat connectivity: a multispecies approach and comparison of resistance surfaces. PLoS ONE.

[102] Macdonald DW (2020). Predicting biodiversity richness in rapidly changing landscapes: Climate, low human pressure or protection as salvation?. Biodivers. Conserv..

[103] Herrero S, Smith T, DeBruyn TD, Gunther K, Matt CA (2005). From the field: brown bear habituation to people—safety, risks, and benefits. Wildl. Soc. Bull..

[104] Skuban M (2017). Effects of roads on brown bear movements and mortality in Slovakia. Eur. J. Wildl. Res..

[105] Findo S, Skuban M, Kajba M, Chalmers J, Kalaš M (2019). Identifying attributes associated with brown bear (*Ursus arctos*) road-crossing and roadkill sites. Can. J. Zool..

[106] Watson JEM (2016). Persistent disparities between recent rates of habitat conversion and protection and implications for future global conservation targets. Conserv. Lett..

[107] Boitani L, Ciucci P, Corsi F, Dupre E (1999). Potential range and corridors for brown bears in the Eastern Alps. Italy. Ursus.

[108] Cushman SA, McKelvey KS, Hayden J, Schwartz MK (2006). Gene flow in complex landscapes: testing multiple hypotheses with causal modeling. Am. Nat..

[109] Mohammadi A (2018). Road expansion: a challenge to conservation of mammals, with particular emphasis on the endangered Asiatic cheetah in Iran. J. Nat. Conserv..

