# Supplementary material for: Identifying priority core habitats and corridors for effective conservation of brown bears in Iran
Source: Sci Rep. 2021 Jan 13;11:1044. doi: 10.1038/s41598-020-79970-z (PMC7806652; doi:10.1038/s41598-020-79970-z)
Supplement: Supplementary file 1 — Supplementary Information. [file 41598_2020_79970_MOESM1_ESM.pdf]

Identifying Priority Core Habitats and Corridors for Effective Conservation of Brown Bears in Iran

A. Mohammadi[1*], K. Almasieh[2], D. Nayeri[3], F. Ataei[4], A. Khani[5], J. V. López-Bao[6], V. Penteriani[6], S. A. Cushman[7]

1. Department of Environmental Science and Engineering, Faculty of Natural Resources, University of Jiroft, Jiroft, Iran.

2. Department of Nature Engineering, Agricultural Sciences and Natural Resources University of Khuzestan, Mollasani, Iran.

3. Department of Environmental Sciences, Faculty of Natural Resources, University of Tehran, Karaj, Iran.

4. College of Environment, Karaj, Iran.

5. Khorasan-e Razavi Provincial Office of the Department of the Environment, Mashhad, Iran.

6. Research Unit of Biodiversity (UMIB, CSIC/UO/PA), 33600 Mieres, Spain.

7. USDA Forest Service Rocky Mountain Research Station, 2500 S. Pine Knoll, Flagstaff, AZ, USA.

[*]Corresponding author: Alireza Mohammadi

Email: armohammadi1989@gmail.com

Fax: (034)-43347065 Phone: (034)-43347061

Table S1- Detecting optimal variables for habitat modeling of brown bear based on the maximum of AUC and the minimum of AIC.

	Regularization multiplier	AICc	AUC-test	AUC-diff	Number of selected variables	Selected variables
Max AUC	1.5	4845.61	0.885	0.008	8	Roughness-BIO1-BIO12-Orchard density- Forest density, Grassland density- Distance to rivers- human footprint
Min AIC	3	4835.03	0.875	0.016	7	Roughness-BIO1-BIO12-Orchard density- Forest density, Grassland density-NDVI, Distance to rivers- human footprint

Table S2 - Variance inflation factor (VIF) of variables chosen by MaxentVariableSelection in habitat modeling of the brown bear in Iran

Variables chosen by MaxentVariablesSelection	VIF
Roughness	1.29
BIO1	1.34
BIO12	1.89
Orchard density	1.03
Forest density	1.76
Grassland density	1.11
NDVI	2.37
Distance from rivers	1.5
Human footprint	1.46

Table S3 - Variable contribution in the habitat modeling of the brown bear in Iran at six spatial scales. Bolded values are the most important variables.

**Spatial scales**	Roughness	BIO1	BIO12	Orchard density	Forest density	Grassland density	NDVI	Distance to rivers	Human footprint
1km	**25.5**	**12**	6	3.2	**14.1**	5.2	**20.2**	7.9	5.9
2km	**25.2**	**10.8**	7.6	2.1	**16.4**	3.2	**21.3**	8.1	5.3
4km	**23.4**	6.6	**8.5**	3.1	**30.1**	4.1	**14.3**	5.6	4.3
8km	**25.2**	5.2	**8.2**	2.9	**30.1**	3.2	**13.2**	4.7	7.3
16km	**26.8**	8	5.9	3.2	**28.1**	3.7	**11.3**	4.2	**8.8**
32km	**19.9**	6.1	5.9	**8.9**	**40.1**	2.4	**10.1**	3.8	2.8

Table S4 - Core areas and number of different protected area categories inside identified brown bear core habitats in Iran.

Core number	Core area	Number of No-Hunting Areas	Number of Protected Areas	Number of Wildlife Refuges	Number of National Parks
1	37030.18	7	11	0	1
2	38881.39	16	10	0	3
3	6279.49	1	2	0	0
4	4786.76	0	2	0	0
5	5843	0	1	0	0
6	3028.42	0	2	0	0
7	2120.02	0	1	0	0
8	1393.69	1	0	0	0
9	2161.95	0	1	0	0
10	166.65	0	0	0	0
11	1191.41	1	0	0	0
12	1442.65	0	0	0	0
13	232.81	1	0	0	0
14	1011.83	1	0	0	0
15	124.46	1	0	0	0
Total	23503.65	29	30	0	4

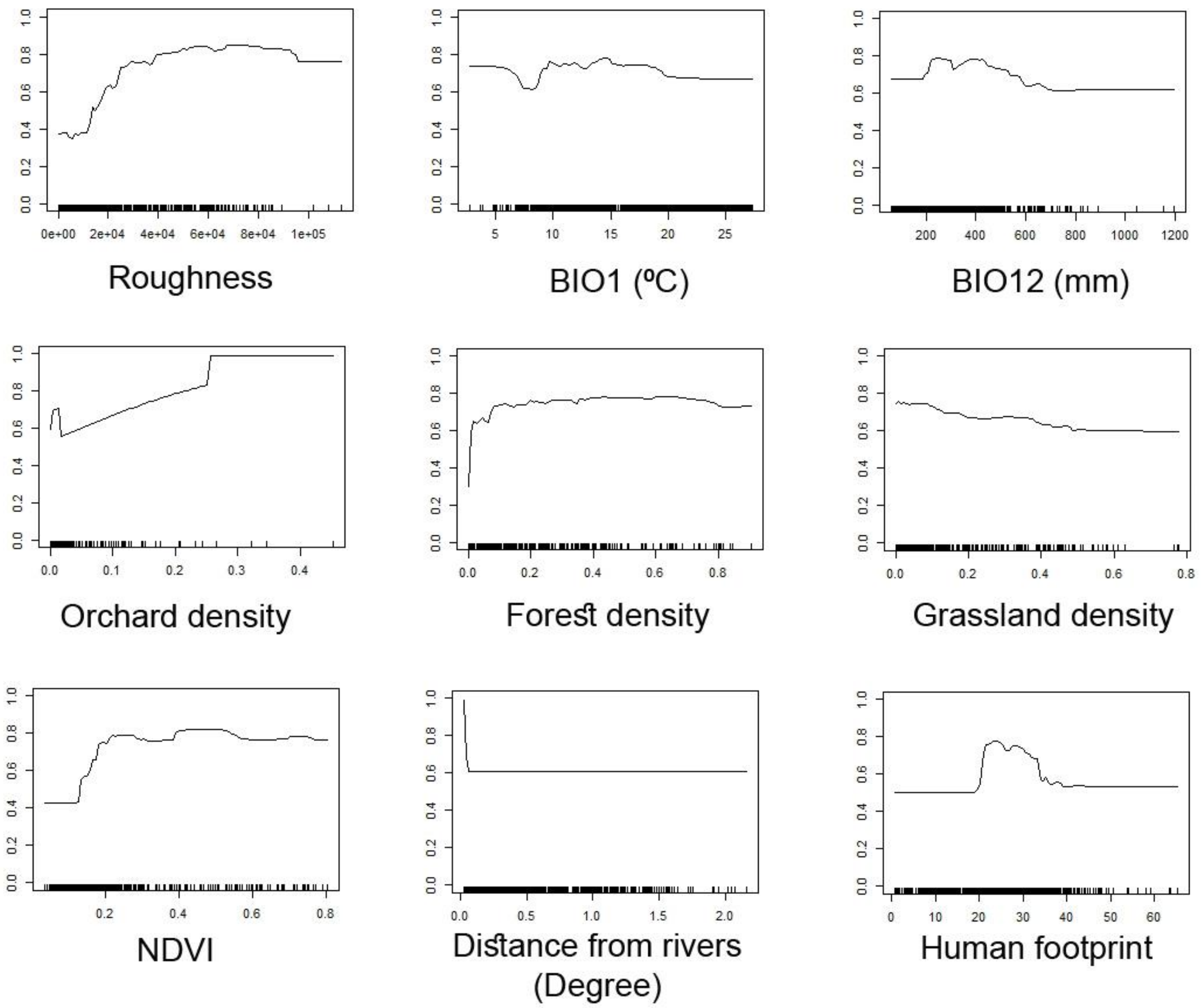

Figure S1-Response curves of presence points to the environmental variables in habitat modeling of the brown bear in Iran.

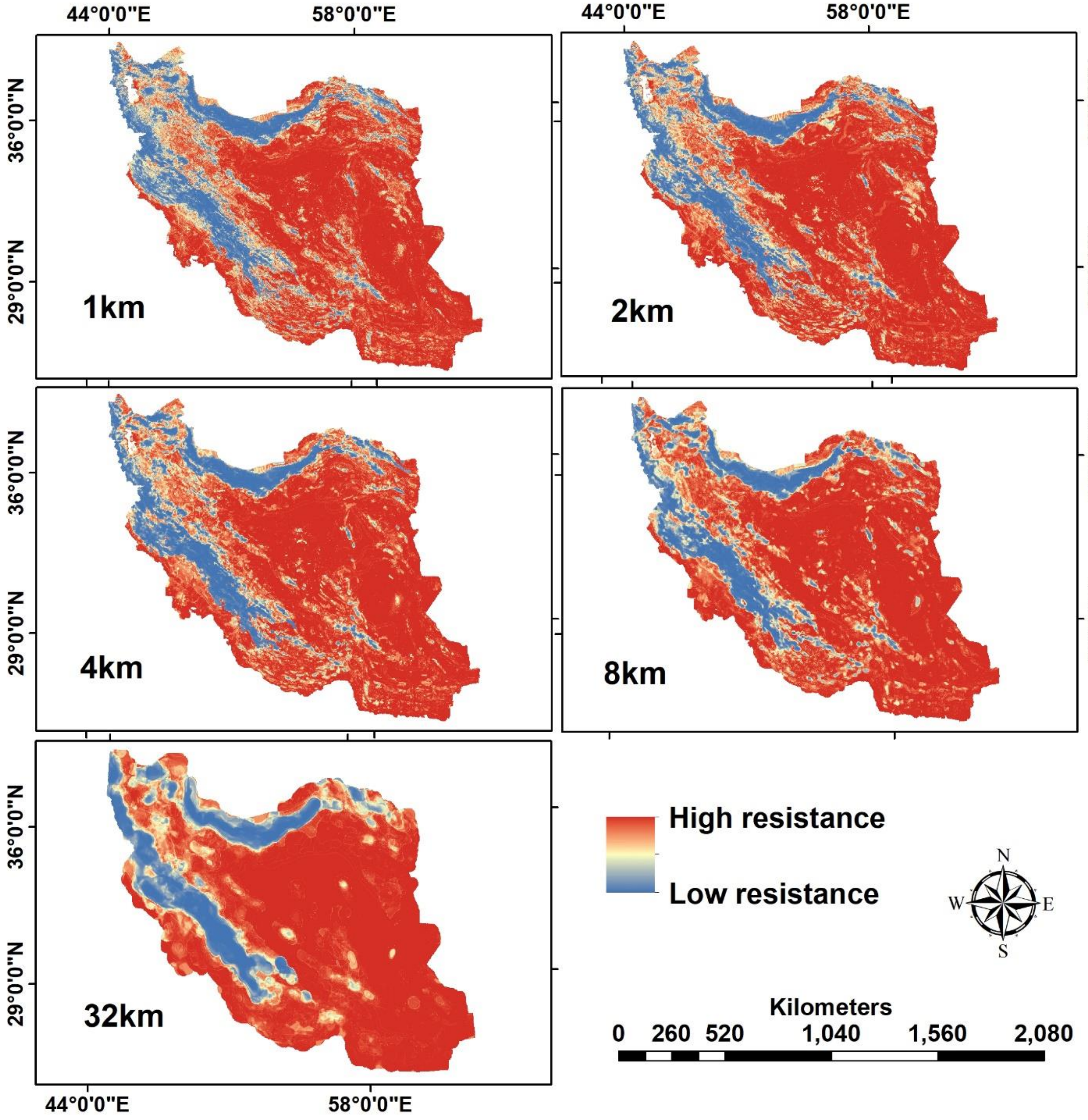

Figure S2 - Resistance maps of the brown bear at five spatial scales in Iran.
